# The Effect of Age on Fracture Risk: A Population-Based Cohort Study

**DOI:** 10.1155/2016/5071438

**Published:** 2016-05-31

**Authors:** Wenbin Liang, Tanya Chikritzhs

**Affiliations:** National Drug Research Institute, Curtin University, GPO Box U1987, Perth, WA 6845, Australia

## Abstract

*Aim*. To precisely estimate the effect of age on the risk of fracture hospitalisation among the Western Australia population over the life course.* Methods*. This population-based cohort study used hospital data on fractures for the period January 1991 to January 2013 among Western Australians born between 1915 and 1990.* Results*. The average incidence rates (per 10,000 person-years) of fracture hospitalisation (95% confidence interval) were 50.12 (49.90, 50.35), 55.14 (54.82, 55.48), and 45.02 (44.71, 45.32) for both males and females, males only, and females only, respectively. The age-specific rate of fracture hospitalisation (in natural logarithm form) in adults (>18 years) was well predicted by age at its 1st, 2nd, and 3rd power in males with an adjusted *R*-squared of 0.98 and *p* < 0.001. For females, the trend was also well predicted by its 1st and 2nd powers (the 3rd power term of age was removed due to its *p* value > 0.8) with an adjusted *R*-squared of 0.99 and *p* < 0.001.* Conclusions*. Overall trends in age and gender specific risk of fracture among the Western Australian population were similar to estimates reported from previous studies. The trend in fracture hospitalisation risk over the life course can be almost fully explained by age.

## 1. Introduction

Injuries are a major cause of morbidity and mortality in both developing and developed countries, while fractures account for a substantial proportion of injuries [1–3]. Fractures not only cause temporary or permanent physical disability but may also impair the overall life quality and mental health of affected individuals [4, 5] and lead to significant burden on healthcare systems as well as family carers [1, 5–7]. Age and gender are important factors that influence the risk of fractures [8]. A number of European epidemiological studies have focused on the effects of age and gender among adult [9–14] and child [15–18] populations. There are limited data available on the overall lifetime incidence rate of fracture among the general Australian population [19, 20], and the majority of Australian studies have focused on site specific fractures [20–22]. In this study, we aim to investigate the effect of age and gender on the risk of fracture among the Western Australian population and apply age to model fracture risk over the life course with good precision.

## 2. Method

This population-based cohort study used hospital separation data on injuries and fractures due to injuries for the period January 1991 to January 2013 among Western Australians (WA) born between 1915 and 1990 (participants are 75–0 years old at baseline). Data were extracted from the WA Hospital Morbidity Data System by the WA Department of Health. The WA Hospital Morbidity Data System included separations from all WA public and private acute hospitals [23]. Hospitalisation records were coded using ICD-9 between 1990/91 and 1998/99 (fiscal year) and ICD-10 classification system from 1999/00. In this study primary diagnosis, codes including ICD-9 codes (800–829) and ICD-10 codes (S12, S22, S32, S42, S52, S62, S72, S82, S92, T02, T08, T10, and T12) were used to identify fracture cases, whereas External Causes of Injury and Poisoning (ICD-9) and External Causes of Morbidity and Mortality (ICD-10) were used to define hospitalisations due to injuries (rather than pathologic causes). Annual estimates of WA gender and age-specific residential populations over the observation period were provided by the Australian Bureau of Statistics (ABS).

## 3. Data Analysis

In the descriptive analysis, overall and age-specific incidence rates of fracture were estimated by age between 0 and 85 years in 2-year intervals, separately for males and females. The age-specific incidence rates were then used to compute the cumulative risk of having at least one fracture hospitalisation for males and females. The risk of hospitalisation due to any injury (including nonfracture cases) was also described using gender and age-specific incidence rates. To investigate how the effects of gender may change over the lifetime, age-specific female-to-male incidence rate ratios were estimated. Log-linear models with age, age to the power of 2, and age to the power of 3 as predictors were fitted based on the age-specific risk of fracture for ages 18 to 85, separately for males and females.

## 4. Results

Overall 193,434 cases of fracture hospitalisation arose from 38,591,627 person-years of observation. The average incidence rates (per 10,000 person-years) of fracture hospitalisation (95% confidence interval) were 50.12 (49.90, 50.35), 55.14 (54.82, 55.48), and 45.02 (44.71, 45.32) for both males and females, males only, and females only, respectively. The incidence rates (per 10,000 person-years) for overall injury hospitalisations (95% confidence interval) were 151.24 (150.85, 151.63), 168.82 (168.2, 169.40), and 133.37 (132.85, 133.89) for both males and females, males only, and females only, respectively. The overall fracture-to-injury risk ratio was about 1 to 3.

**Figure 1:**
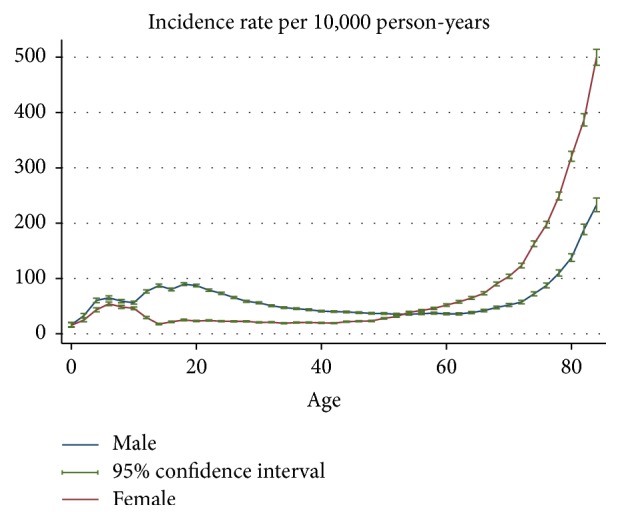
Gender-age-specific incidence rates of fracture hospitalisation.

**Figure 2:**
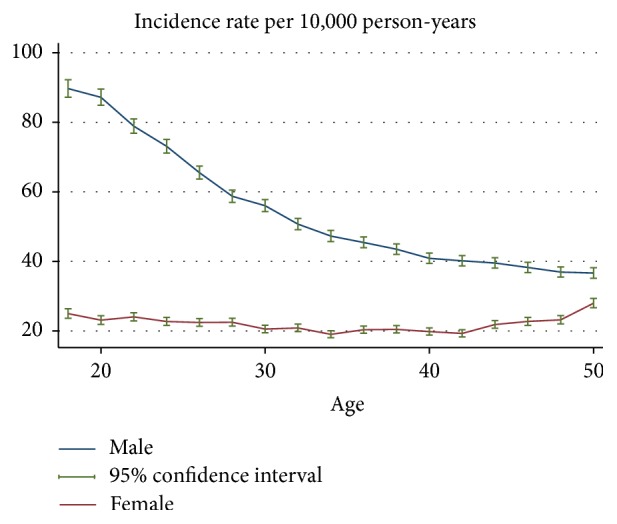
Gender-age-specific incidence rates of fracture hospitalisation for the age between 18 and 50 years.

**Figure 3:**
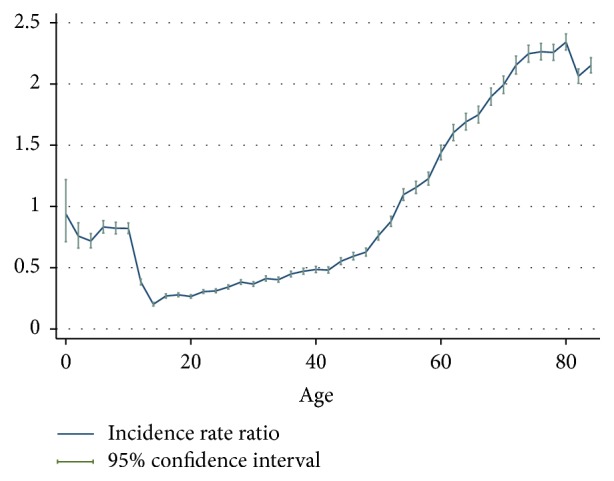
Age-specific female-to-male incidence rate ratios of fracture hospitalisations.

The gender-age-specific incidence rates of fracture hospitalisation are shown in Figure 1.  Figure 2 provides a close-up illustration of the risk pattern for ages 18–50 years. Overall, the risk of fracture increased continuously up to 8 years of age and remained unchanged between 6 and 11 years for both genders. From the age of 12, the risk of fracture increased gradually until the age of 19 in males, whereas the risk of fracture decreased over adolescent period for females. The risk of fracture declined in males from age 20 years through to the late 40s; among females, however, the risk of fracture began increasing from about the age of 42 years. Up to the age of 54 years, the incidence rate of fracture was consistently higher for males compared to females; thereafter, female rates exceeded male rates and remained higher to the end of the observation period. Accordingly, the age-specific female-to-male incidence rate ratios of fracture increased from the age of 20 (Figure 3). The pattern of age-specific injury hospitalisation rates appeared to be similar between the two genders, except for ages 55 to 65, when the risk of injury continuously increased in females but remained largely unchanged in males (Figure 6).

**Figure 4:**
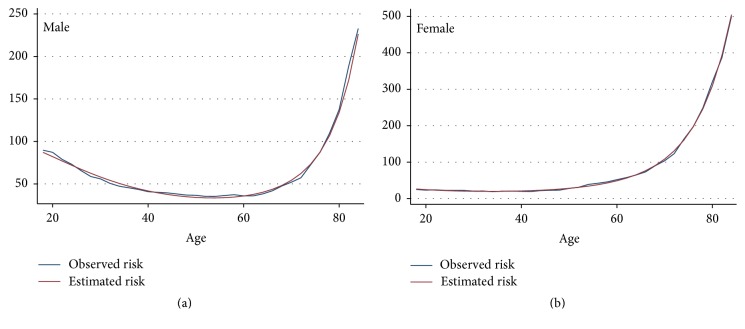
Comparison between observed gender-age-specific incidence rates of fracture hospitalisation and estimates from log-linear model (per 10,000 person-years).

The age-specific rate of fracture (in natural logarithm form) in adults (>18 years) was well predicted by age at its 1st, 2nd, and 3rd power in males with an adjusted *R*-squared of 0.98 (*p* < 0.001). For females, the trend was also well predicted by its 1st and 2nd powers (the 3rd power term of age was removed due to its *p* value > 0.8) with an adjusted *R*-squared of 0.99 (*p* < 0.001) (see Table 1). Comparison between the observed incidence rates of facture and estimates from the model are shown in Figure 4.

**Figure 5:**
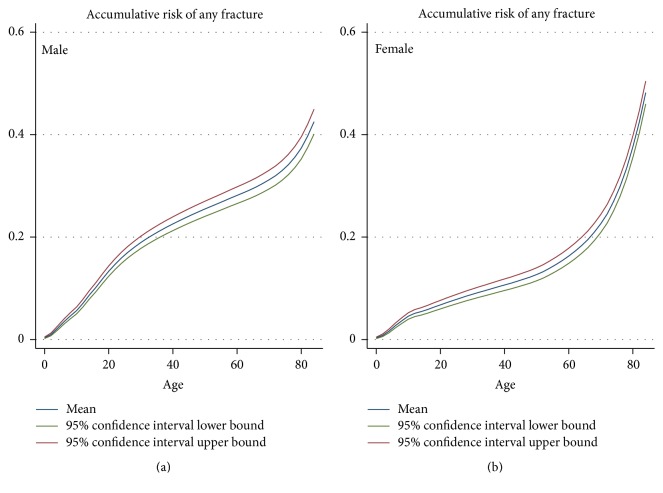
Cumulative risk of having any fracture hospitalisation since birth.

For males and females, respectively, the cumulative risk of having at least one fracture hospitalisation due to injury was 11.8% and 6.3% by the age of 18 years, increased to 23.8% and 11.4% by the age of 45 years and increased again to 37.4% and 37.6% by 80 years (Figure 5).

## 5. Discussion

**Figure 6:**
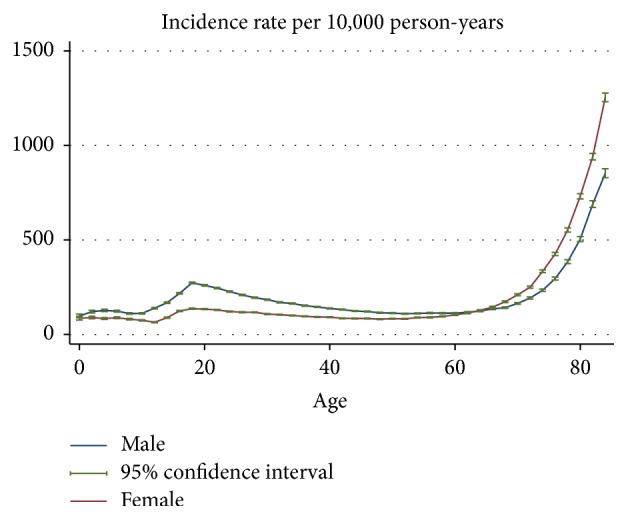
Gender-age-specific overall injury hospitalisations rates.

This study provided a detailed picture of the risk of fracture hospitalisations among the Western Australia population. Overall trends in age and gender specific risk of fracture were similar to observations from previous studies conducted on other populations [9–14, 20, 24]. The relative increase in fracture risk among females compared to males throughout the 20s and 30s (Figure 3) was also evident although this has not been explored in detail in some of the earlier studies [12, 13, 19]. This increasing trend of female-to-male fracture incidence rate ratios appears to be driven by a decrease in risk for males and stable risk for females in that age period (Figure 2). However, the age-specific risk of all injuries appeared to decrease at a similar rate for the two genders (Figure 6), and therefore the increase in female-to-male incidence rate ratio in the 20s and 30s is unlikely to be entirely due to reductions in involvement of injury-related activities among males as they mature. The observed gender difference in the age-specific trend of fracture over this age period may be due to sex-specific changes in musculoskeletal fitness. This study also showed that the changes in the probability of fracture in adults over the life course can be expressed as a function of age. This suggests that the variation in fracture risk at different life stages is driven by risk factors (causes) that are determined or strongly associated with age. There is good evidence suggesting that the progression of osteoporosis [6, 25], changes in musculoskeletal fitness [2, 26], probability of involvement in injury-related behaviours [26, 27], and having certain health conditions [8, 28] are dependent on age; however, it is unclear what other factors may underpin the strong association between fracture risk and age, and further research is required.

This study has some limitations. The analyses relied entirely on hospitalisation cases, which do not include fractures that are treated by outpatient services (such as at Emergency Departments and GP clinics) and which may be relatively less severe. In addition, the study is unable to distinguish first ever and secondary fractures. However, strengths of the study include its very large population-based sample, long follow-up period, application of a number of statistical methods, and the provision of detailed estimates of the absolute risks and cumulative risk of facture from birth to 85 years. In addition, this study showed that the trend in fracture risk over the life course can be almost fully explained (i.e., predicted) by age.

## 6. Conclusion

Overall trends in age and gender specific risk of fracture among the WA population were similar to estimates reported from previous studies. There is a relative increase in fracture risk among females compared to males throughout their 20s and 30s which could not be explained by reductions in injury-related activities among males. The trend in fracture risk over the life course can be almost fully explained by age.

## Figures and Tables

**Table 1:** log-linear model of age-specific fracture hospitalisation rates.

Gender	Model and estimated coefficients
Male	ln⁡(risk of fracture) = 4.648261 + 0.0158851 × age − 0.0017527 × age^2^ + 0.0000198 × age^3^
Female	ln⁡(risk of fracture) = 4.405889 − 0.0843678 × age + 0.0012531 × age^2^

ln: natural logarithm.
